# Exploring the effects of climate-related financial policies on carbon emissions in G20 countries: a panel quantile regression approach

**DOI:** 10.1007/s11356-021-15655-y

**Published:** 2021-09-03

**Authors:** Paola D’Orazio, Maximilian W. Dirks

**Affiliations:** Institute of Macroeconomics, Faculty of Economics and Management, Ruhr-Universität Bochum, Universitätsstraße 150, 44801 Bochum, Germany

**Keywords:** Financial development, Climate-related financial policies, Carbon dioxide emissions, Climate risks, Green finance

## Abstract

This paper studies the effects of financial development, economic growth, and climate-related financial policies on carbon emissions for G20 countries. The focus is particularly on financial policies implemented to scale up green finance and address climate-related financial risks from 2000 to 2017 and represent this paper’s value added. The empirical results obtained by relying on the panel quantile regression approach indicate that the impacts of the different explanatory variables on carbon emission are heterogeneous. Specifically, the effect of the stock of short-term financial policies on carbon emissions is negative, and its effect becomes smaller at higher quantiles. The stock of long-term policies also shows significant negative coefficients, but its impact is stronger for higher quantiles. No significance is reported for the lowest quantile. Financial development contributes to improving environmental quality, and its impact is larger in higher emission countries. Energy consumption increases carbon emissions, with the strongest effects occurring at higher quantiles. Our results also support the validity of the EKC relationship and positive effects of GDP and population on high carbon emissions levels. Estimation results are robust to alternative model specifications and after controlling for the role played by adopting international climate change mitigation policies as proxied by the adoption of the Kyoto Protocol.

## Introduction

Global warming has become one of the most severe and pressing issues because of the devastating consequences of environmental degradation on humanity and economic systems globally. The human effect on climate change is also widely reported, and carbon emissions are now considered the highest in history (Nagelkerken and Connell 2015). Carbon dioxide (CO_2_) is the most important greenhouse gas implicated in global warming (Scheffer et al. 2006; Solomon et al. 2009). Its accumulation in the atmosphere beyond certain limits can lead to irreversible impacts, which will be challenging to tackle at later stages (IPCC 2014, 2018).

At the international level, several efforts are put forward to mitigate climate change’s adverse effects by reducing carbon emissions. The UN Paris Agreement sets the goal of keeping global warming well below 2 °C and as close as possible to 1.5 °C above pre-industrial levels. To comply with this objective, countries should reduce emissions to almost zero by 2050. Nevertheless, the special report of the Intergovernmental Panel on Climate Change (IPCC 2018) on the global temperature goals shows that the gap between current trends and emission reduction targets set by countries through their nationally determined contributions (NDCs) is widening and leading to somewhere between 3 and 4 °C of warming (den Elzen et al. 2019). This scenario is consistent with what has been defined as a “Hothouse Earth” pathway (Steffen et al. 2018). Indeed, the most recent evidence suggests that global carbon emissions have been increasing despite the efforts to reduce them (Peters et al. 2020). In 2020, the International Energy Agency (IEA) reported that global energy-related CO_2_ emissions flattened in 2019, following 2 years of increases. According to the analysis, this resulted mainly from a sharp decline in CO_2_ emissions from the power sector in advanced economies, thanks to the expanding role of renewable sources (primarily wind and solar PV), fuel switching from coal to natural gas, and higher nuclear power output (IEA 2020). However, according to Le Qúeŕe et al. (2019), to limit global warming to below 2 °C by the year 2100, greenhouse gas emissions would have to decrease by one to two billion tons every year.

Because of the natural, social, and economic disruption it risks generating, the research community from different disciplines has agreed on the compelling need to tackle climate change (Stern et al. 2006; Heller and Zavaleta 2009; Rockstr¨om et al. 2009; Steffen et al. 2018; Dasgupta 2021). As CO_2_ is the primary responsible for global warming, the determinants of CO_2_ emissions have been largely studied. Among the main drivers, economic growth is usually examined because it promotes economic development and progress. Still, it is detrimental to the environment. Therefore, existing literature has investigated the nexus between economic growth and the environment, popularly known as the Environment Kuznets Curve (EKC). Besides the EKC relationship, some researchers focused on the role of democracy and renewable energy in reducing carbon emissions (Adams and Acheampong 2019); others looked at the impact of democracy, globalization, and urbanization (Wang et al. 2018); the role of financial stability and energy consumption (Nasreen et al. 2017) and a broad stream of literature studied the effects of financial development (see G¨ok 2020, for a recent review). The latter is particularly relevant because both capital markets and the financial sector play a crucial role in delivering the necessary investments in low-carbon technologies to achieve green structural change.

However, in our view, a closer look at the dynamics of CO_2_ emissions and climate change, and the development of climate-related financial policies in the past decades suggests the existence of a more complex picture that needs to be investigated. First, the implementation of adaptation and mitigation strategies is related to developing green technologies whose diffusion is constrained by several “barriers,” such as costs, lack of competencies and knowledge, market structure, and lack of financial resources (D’Este et al. 2012). The latter is particularly relevant and is motivated by the fact that eco-innovation requires long-term financial capital, which is, by definition, riskier and, therefore, more expensive than standard “non-green” innovation (Mazzucato and Semieniuk 2018; D’Orazio and Valente 2018). Second, although positive trends of green finance development have been detected in the past years, the flow of financial resources is insufficient to close the “green finance gap” (Buchner et al. 2017; IEA 2018; Geddes et al. 2018; D’Orazio and L¨owenstein 2020). As a result, the existing green finance volumes fall short to meet the 2 °C scenario called for by the Intergovernmental Panel on Climate Change (IPCC 2018), and a green structural change is difficult to achieve. Overall, this evidence suggests that, when studying the dynamics of carbon dioxide emissions in the past decades, it is essential to account for novel variables. In particular, we claim that it is necessary to include climate-related financial policies (CRFPs), which are characterized by two main aims, i.e., to scale up green finance^1^ and tackle physical, and transition risks for the financial sector (Carney 2015) and are promoted mainly by central banks and financial regulators (Batten et al. 2016; Campiglio et al. 2018; D’Orazio and Popoyan 2019; Krogstrup and Oman 2019). Drawing on recent literature contributions that point out the crucial role of central banks and financial regulators in addressing the climate challenge (see Carney 2015; Batten et al. 2016, among others) and considering the two main goals of climate-related financial policies as highlighted above, the hypothesis behind our study is that the adoption of climate-related financial policies codifies a country’s policy ambition concerning CO2 emissions, implying that a higher number of policies lead to lower CO2 emissions. On the one hand, the rationale is that some of these policies can directly promote the allocation of financial capital to sustainable, green, and nonpolluting activities, thus promoting a low-carbon transition. On the other hand, other types of policies can create favorable conditions for green and sustainable investments by encouraging, for example, the disclosure of exposure of financial institutions to “brown/polluting” assets or the adoption of a clear economic activities taxonomy (i.e., sustainable, non-sustainable, neutral), thus contributing to the progressive decarbonization of the economy and low carbon transition (Wimbadi and Djalante 2020). Moreover, in our view, the adoption of climate-related stress tests or climate-aligned financial risk management measures could also have a role in curbing CO2 emissions. Once they signal the existence of relevant climate risks to the financial sector, they encourage financial institutions to gradually reduce their exposure to “climate-sensitive” assets to decrease the size of potential losses deriving from the materialization of extreme climate events deriving from global warming.

Within the context of climate change mitigation, the effect of these policies on CO_2_ emissions stands out as a complex issue due to different new financial regulations that G20 countries have adopted in the past decades (see the “Data” section for a review). However, despite their importance in shaping the climate-related financial landscape, the link between climate-related financial policies and CO_2_ emissions is still under-investigated (De Haas and Popov 2019).

Against this backdrop, the contribution of this paper to the literature is threefold. First, to the best of our knowledge, climate-related financial policies have not been considered in any empirical analysis of the determinants of CO_2_ emissions. Our study investigates this issue for the first time and thus provides a primary contribution to understanding their role in affecting environmental quality (proxied by CO_2_ emissions).^2^ Second, this study applies a panel quantile regression (PQR) approach to explore the effect of climate-related financial policies, financial development, and economic growth on the CO_2_ emissions. Applying the PQR approach will allow us to provide more detailed results than the standard ordinary least squares (OLS) approach and discuss the heterogeneity characterizing countries’ experiences. Third, the study’s focus on G20 countries is important because they represent the leading global economies and offer a broad sample of developed and developing countries. Furthermore, they are the most significant contributors to global carbon emissions. Hence, we deem it relevant to study the role of climate-related financial policies from 146 the G20 perspective.

The remainder of the paper is organized as follows. The “Literature review” section offers a review of the literature and puts our study in context. Data are described in the “Data” section and the methodology is presented in the “Method” section. The “Empirical results and discussions” section provides the empirical results and discussions, and, finally, the “Conclusions and policy implications” section offers concluding remarks and discusses the policy implications of the investigation.

## Literature review

### Economic growth, financial development, and environmental quality

After the seminal contribution by Grossman and Krueger (1995), academic research has focused on the effects of growth on CO_2_ emissions. The relationship between the measure of environmental degradation, i.e., CO_2_ emissions, and per capita income is defined as the Environmental Kuznets Curve (EKC).

The EKC hypothesis states that an inverted U-shaped relationship between environmental degradation and economic growth exists: environmental degradation increases at the initial stage of economic growth; as the economy grows, environmental degradation falls, and environmental quality improves. The evidence on the EKC is, however, still mixed. According to some researchers, the EKC relationship is not valid because it is observed that environmental degradation is monotonically increasing with economic growth (see Stern 2004; Alkhathlan and Javid 2013; Farhani and Ozturk 2015, among others). Others find instead support for the EKC hypothesis (see He and Richard 2010; Apergis and Payne 2010; Apergis and Ozturk 2015; Jebli et al. 2016, among others). Therefore, it is essential to test the EKC hypothesis’s validity when designing appropriate policy tools to fight against global warming and protect the environment.

Moreover, when discussing the relationship between economic growth and CO_2_ emissions, it is important to consider financial development. The rationale for this is that financial development enhances economic growth, which requires, among others, more energy consumption, resulting in pollution and environmental degradation. While financial development is claimed to have significant implications for the environment, its impact on carbon emissions remains contentious.

Two main opposing views about the impact of financial development on the environment can be distinguished. Some scholars believe that financial development improves the environment’s quality by reducing carbon emissions. They argue that financial development can facilitate more financial resources at a lower cost, thus increasing financing for environmental projects (see Tamazian et al. 2009; Acheampong 2019, among others), and that financial development may provide enough incentives for firms to lower their CO_2_ emissions (Lanoie and Roy 1997; Dasgupta et al. 2001). Some researchers also maintain that financial development provides developing countries with more opportunities for advanced technologies, which implies increased energy efficiency and sustainable and environmentally friendly production, thus reducing CO_2_ emissions (Claessens and Feijen 2007). According to others, financial development contributes instead to environmental degradation: it increases households’ consumption and firms’ production and accelerates economic growth, resulting in polluting activities and thereby causes an increase in emissions. Among others, Zhang (2011) found that financial development is one of the factors responsible for increasing the level of CO_2_ emissions. Financial development also contributes to a rise in energy consumption and CO_2_ emissions in Sub-Saharan African countries, as discussed in Al-Mulali et al. (2012). As noted by G¨ok (2020), the difference in the estimated effect depends on the financial development indicator used (see Acheampong 2019, on this point), the econometric technique employed, selected countries, country group, region, or provinces involved, and period considered in the investigation.

Overall, by considering existing literature on the role of financial development on CO_2_ emissions, it seems that different impacts are observed for different countries or groups of countries (G¨ok 2020). On the one hand, studies considering African countries or focusing on individual countries such as Brazil, Greece, India, Indonesia, Nigeria, Pakistan, Portugal, Saudi Arabia, Sri Lanka, Tunisia, Turkey, USA, and Vietnam tend to report a significant positive impact on the effect of financial development on CO_2_ emission. On the other hand, studies analyzing emerging markets, Asia, Commonwealth of Independent States (CIS), European Union (Park et al. 2018), Gulf Cooperation Council (GCC), Next 11, APEC (Zaidi et al. 2019), and OECD countries (Lee et al. 2015) or focusing on individual countries such as Bangladesh (Khan et al. 2018), China, Iran, Malaysia, and Russia tend to report a significant negative impact on the effect of financial development on CO_2_ emission.

### Green financial development: climate-related financial policies and environmental quality

Central banks and financial regulators have been particularly engaged in climate-related financial policymaking in the past decades. Although they cannot substitute for an adequate climate policy (Lane 2019; Weidmann 2020), it is now widely acknowledged that they have to take action to scale up green finance and adopt regulations to address climate-related financial risks. The rationale for this is that (i) climate change affects monetary policy and financial regulation (Batten et al. 2016; Campiglio 2016; D’Orazio and Popoyan 2019; Chenet et al. 2021) and (ii) financial actors play an essential role in the global economy (Rogoff 1999; Mazzucato and Penna 2016; Wang 2017; Geddes et al. 2018).

Regarding their green finance action, they can redirect financial flows towards activities that protect natural capital and positively affect the environment (Galaz et al. 2015). This policy action is also aligned to the Paris Agreement goal (Article 2.1c) of “making finance flows consistent with a pathway towards low greenhouse gas emissions and climate-resilient development” (COP 2015). Compared with traditional finance, green finance emphasizes environmental interests; it regards environmental protection and the effective use of resources as important criteria for measuring the effectiveness and ultimately realizes sustainable development and promotes economic growth (Yadav and Pathak 2013; Steckel et al. 2017; Sachs et al. 2019). Therefore, like financial development, the development of green finance can also promote economic growth. Regarding action taken against climate risks, their effort is crucial as climate change poses threats to the conduct of monetary policy because of its effects on supply price shocks, market volatility, and economic growth, which are related to inflation through credit spreads, saving rates, and real interest rates (Coeuŕe 2018; Monnin 2018; Schnabel 2020). Moreover, climate-related financial risks (CRFRs), namely, physical, transition, and liability risks represent a threat to financial stability (Carney 2015). Climate-related financial risks can cause credit risks, market risks, liquidity risks, and insurance risks because of financial losses, destruction of production capital, the decline in profitability of exposed firms, and stranding of assets related to climate-relevant sectors (e.g., fossil fuels and mining) (Batten et al. 2016; Elderson 2018). Central banks and regulators are thus required to assess financial institutions’ performance and report how they account for environmental and social issues and provide guidance and requirements regarding how financial institutions impact ecosystems (Carney 2015; Scholtens 2017; HLEG 2018; ECB 2020).

Several policies are suitable for scaling up green finance or address climate risks, thus contributing to decarbonization and low-carbon transition. We define them as climate-related financial policies (CRFPs) throughout the paper, and we distinguish among four categories, namely, (i) climate-related prudential regulations; i.e., measures such as governance and risk management measures, climate-related stress tests, and climate-related risk disclosure aimed at the banking sector; (ii) promotional credit measures; (iii) green financial principles; i.e., policies aimed at “creating green financial markets,” such as green finance principles and green taxonomy; and (iv) other climate-related disclosure requirements aimed at non-financial institutions, insurance companies, and pension funds.

In the first category, several prudential measures existing under the Basel III framework can be used to meet the Paris Agreement goals (see D’Orazio and Popoyan 2019, for a review). Among others, leverage ratios, countercyclical capital buffers, and risk weights applied to banks’ assets, such as the Green Supporting Factor (GSF) or the Brown Penalising Factor (Schoenmaker and Van Tilburg 2016), could be implemented. Following the proposal of a GSF by the European Commission (Dombrovskis 2017), green capital regulations have been discussed in the past years, with many analyses pointing to significant risks deriving from its potential implementation. Among others, threats to financial stability are emphasized, considering that green assets are not risk-free. Sectoral leverage ratios could also be implemented to limit the financial sector’s exposure to brown assets, thus addressing potential threats from a low-carbon transition. Other measures relate to climate-related stress tests^3^ that provide useful information to policymakers regarding the financial system exposure to climate-related risks (see, e.g., Vermeulen et al. 2019) and their results could be used to calibrate and evaluate green macroprudential tools. Other (banking) prudential measures, such as disclosure requirements of the physical, liability, and transition risks associated with climate change, are also relevant to develop a credible green financial system and avoid the so-called “green washing” (TCFD 2018). Moreover, as noted by Jean-St́ephane et al. (2021), disclosure requirements create incentives for financial institutions to cut their holdings related to non-sustainable/polluting investments “both to meet more rapidly climate-related compliance targets, whatever the metrics used, and to make their claimed climate commitments easier to communicate to the public” (Jean-St́ephane et al. 2021, p.2).

Regarding the second category, central banks can also directly promote green-sustainable investments through promotional credit measures, such as green lending quotas and concessional loans to priority and environmentally friendly sectors (Dikau and Ryan-Collins 2017; Volz 2017).

In the third category, sustainability reporting and compliance practices and green taxonomies are included. The former are increasingly considered complementary to risk management practices in dealing with concerns about the adverse consequences of climate change (Ng 2018), while regarding the latter, several experiences are observed internationally. Some emerging economies, such as Bangladesh, China, and Mongolia, have adopted green taxonomies starting from 2015 (BB 2020; FSCM 2019; GFC 2015), while the debate is more recent in the EU. There, the first proposal dates back to 2018 (EC 2018) and has been followed by establishing a Technical Expert Group (TEG) on sustainable finance. The TEG published its final report in March 2020, providing recommendations relating to the design and guidance on how companies and financial institutions can use and disclose against the taxonomy (TEG 2020). Because of political negotiations, the regulation entered into force only in July 2020.^4^

Finally, the fourth category concerns reporting regulations and environmental, social, and governance (ESG) criteria aimed at pension funds, insurance companies, and other non-financial institutions. Reporting regulations on pension funds have become prominent in the debate on the importance of climate risks for institutional investors (Della Croce et al. 2011) and on how to tackle climate change from the financial sector perspective (see Ameli et al. 2019, among others). In particular, after the Paris Agreement, a better understanding of whether institutional investors’ portfolios are exposed to asset stranding and, consequently, financial risks, has become increasingly important for investors, regulators, and the economy at large (Boermans and Galema 2019; Krueger et al. 2020). Regarding ESG criteria, they have been gathering much consideration, both in the economics profession and among policymakers (Wang and Sarkis 2017; Lokuwaduge and Heenetigala 2017; Li et al. 2018; Widyawati 2020). Indeed, ESG metrics and their implementation have become a critical part of business strategy. Although there are risks of greenwashing (Yu et al. 2020), ESG disclosure is becoming relevant because it is found to have positive effects on firms’ performance, besides reducing uncertainty, business risk, and the cost of capital (El Ghoul et al. 2011; Amel-Zadeh and Serafeim 2018; Bhaskaran et al. 2020).

## Data

The following section presents the variables used in the empirical analysis. Their description and sources are summarized in Table 1 and summary statistics are provided in Table 2. Among others, we note that all variables are skewed; this represents another motivation for employing the quantile regression approach to detect the effect of climate-related financial policies on CO_2_ emissions in this paper.

**Table 1:** Variable definitions and data sources. All data are annual over the period 2000–2017

Variable	Abbreviation	Source
CO2 emissions in metric tons in million	CO2	Ritchie and Roser (2020)
GDP (in billion current US-dollar)	GDP	World Bank, World Development Indicators
Squared GDP (in billion current US-dollar)	GDP2	World Bank, World Development Indicators
Population, in million	Pop	World Bank, World Development Indicators
Regulatory quality	RQ	World Bank, World Governance Indicators
Long-term financial policies	LTFP	Individual assessment, public available information from official sources
Short-term financial policies	STFP	Individual assessment, public available information from official sources
Domestic credit to private sector (share of GDP)	Domcredit World	Bank, Global Financial Development Database
Chinn-Ito index	ChinnIto	Chinn and Ito (2010)
Bank return on assets (percentages, after tax)	ROA	World Bank, Global Financial Development Database
Fossil fuel consumption in TWH per capita	FFC	World Bank based on IEA Statistics

**Table 2:** Summary statistics

Variable	Mean	Std. Dev.	Min.	Max.	Skewness	Kurtosis	*N*
CO2 emissions in metric tons in million	1244.389	1915.701	124.381	9599.007	2.81326	10.33977	342
GDP (in billion current US-dollar)	2425.476	3426.546	97.724	19,519.354	2.973936	11.94385	342
Squared GDP (in billion current US-dollar)	1.76e+07	5.42e+07	9549	3.81e+08	4.339691	22.61093	342
Population, in million	221.536	367.277	19.153	1386.395	2.419018	7.239013	342
Long-term financial policies weighted by regulatory quality	0.698	1.727	− 2.605	10.732	2.604342	11.9387	342
Short-term financial policies weighted by regulatory quality	0.068	0.408	− 0.475	3.521	6.463013	48.43763	342
Domestic credit to private sector (share of GDP)	90.174	53.284	9.683	212.269	.2269743	1.882335	312
Chinn-Ito index	0.835	1.43	− 1.917	2.347	− .3234056	1.607863	342
Bank return on assets (percentages, after tax)	0.984	1.07	− 6.697	6.843	− .860239	14.81627	342
Fossil fuel consumption in TWH per capita	35.463	23.907	3.221	94.873	.6237114	2.342099	342

The sample considered in our investigation includes G20 countries, except the European Union, which was excluded because of the unavailability of data for several variables considered in the analysis. The choice to focus on G20 countries is motivated by the fact that they represent the leading global economies and offer a broad sample of developed and developing countries. Furthermore, they are the most significant contributors to global carbon emissions.

The correlation matrix of the data presented in Table 3 shows a positive relationship between GDP and CO_2_ emissions, population and GDP, fossil fuels consumption, and CO_2_ emissions. The outcomes also reveal positive co-movement of GDP and fossil fuel consumption, on the one hand, and domestic credit to the private sector and GDP, on the other hand. A negative correlation is observed for both the long-term and short-term stocks of policies and CO_2_ emissions.

**Table 3:** Cross-correlation table

Variables	(1)	(2)	(3)	(4)	(5)	(6)	(7)	(8)	(9)	(10)
CO2 emissions in metric tons in million	1.000									
GDP (in billion current US-dollar)	0.764	1.000								
Squared GDP (in billion current US-dollar)	0.677	0.950	1.000							
Population, in million	0.667	0.249	0.181	1.000						
Stock of long-term financial policies weighted by regulatory quality	− 0.152	0.086	0.048	− 0.253	1.000					
Stock of short-term financial policies weighted by regulatory quality	− 0.081	− 0.004	− 0.039	− 0.090	− 0.067	1.000				
Domestic credit to private sector (share of GDP)	0.403	0.588	0.484	0.009	0.289	0.080	1.000			
Chinn-Ito index	− 0.116	0.307	0.211	− 0.472	0.410	0.183	0.396	1.000		
Bank return on assets (percentages, after tax)	− 0.017	− 0.131	− 0.039	− 0.014	− 0.201	− 0.078	− 0.371	− 0.298	1.000	
Fossil fuel consumption in TWH per capita	0.111	0.290	0.303	− 0.365	0.230	0.022	0.378	0.513	0.016	1.000

### Dependent variable

As the dependent variable, we consider total CO_2_ emissions (in million metric tons) deriving from fossil fuels’ consumption, i.e., coal, natural gas, and oil and cement. Because CO_2_ is reported to be the primary greenhouse gas responsible for global warming, we use it as an environmental degradation measure. We collected data from the “CO_2_ and Greenhouse Gas Emissions” database, which uses data from Le Qúeŕe et al. (2018) and provides the latest available data (2017) regarding CO_2_ emissions at the G20 level.

**Fig. 1:**
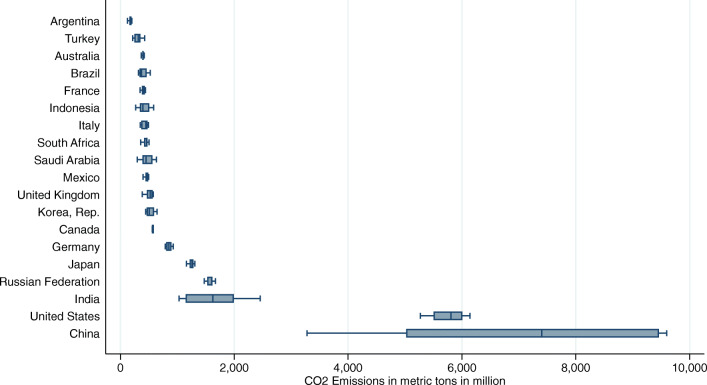
Distribution of CO2 emissions by country (2000–2017)

The heterogeneity concerning the CO_2_ distribution in our sample is shown in Fig. 1, which allows us to visualize the countries that belong to the lowest and highest quantiles. Summary statistics reported in Table 2 show that the distribution of CO_2_ emissions is positively skewed.

### Climate-related financial policies in the G20

Using the taxonomy of policies presented in the “Green financial development: climate-related financial policies and environmental quality” section, we select 93 policies adopted by G20 countries in the period 2000–2017.^5^ Consequently, our CRFP data constitutes a panel of 342 country-year observations (as we consider 19 countries over 18 years).

**Fig. 2:**
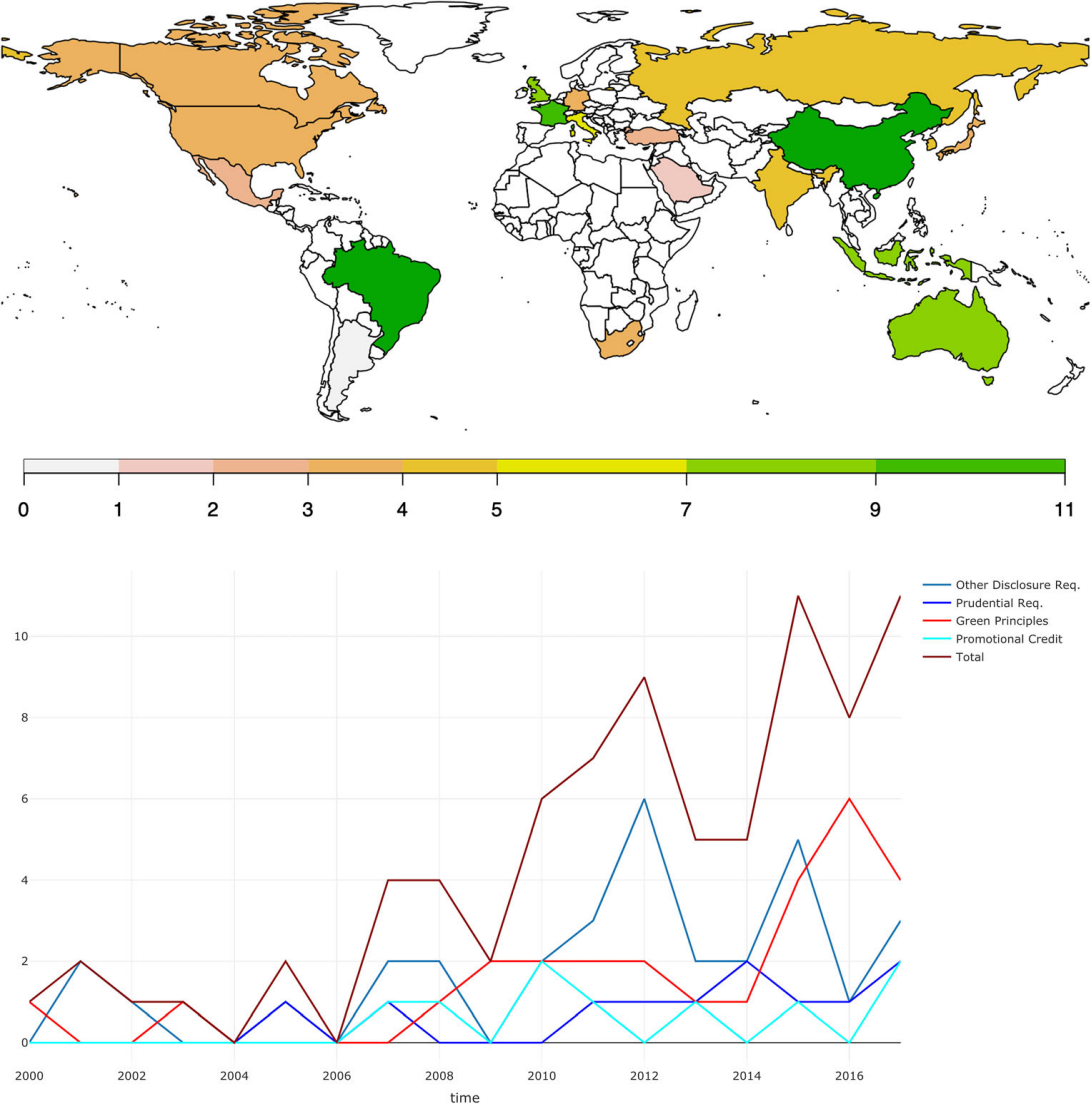
Stock of climate-related financial policies adopted by country at the end of 2017 (top graph). Number of climate-related financial policies adopted per year during 2000–2017 in G20 (bottom graph). Source: authors’ elaboration on data retrieved from D'Orazio (2021)

As shown in Fig. 2 and Table 4, all G20 countries—although to different degrees—have acknowledged the need to adjust national financial architectures and are discussing or have already implemented green financial principles, such as national green finance strategies, and taxonomy of green and brown investments. In particular, our data show that a steady increase over time characterizes the adoption of CRFPs. The rationale for this is that, on the one hand, countries increasingly recognize that climate-related risks such as transition, liability, and physical risks may harm the financial system and financial stability in general; hence, central banks and financial regulators are considering how to integrate them into existing regulatory frameworks (Carney 2015; Thoma and Chenet 2016; Volz 2017; NGFS 2019; D’Orazio and Popoyan 2019). On the other hand, the financial sector’s role in enhancing a low carbon transition by providing adequate financial resources to scale up green finance has got increasing attention in the past decades (Wang and Zhi 2016; Sachs et al. 2019; Hafner et al. 2020; Carney 2021).

**Table 4:** Type of policies adopted by country at the end of 2017. Legend: *GRM*, governance and risk management; *CRST*, climate-related stress test; *DR*, disclosure requirements; *RR*, reserve requirements. Source: authors’ elaboration on data retrieved from D'Orazio (2021)

Country	Macroprudential regulations	Other disclosure requirements	Promotional credit measures	Green financial principles
Argentina		✓		✓
Australia	GRM	✓		✓
Brazil	ICAAP	✓	✓	✓
Canada		✓		✓
China	GRM, CRST, DR, RR	✓	✓	✓
France	GRM	✓		✓
Germany		✓		✓
India		✓	✓	✓
Indonesia	GRM, DR	✓	✓	✓
Italy		✓		✓
Japan		✓	✓	✓
Mexico	GRM, DR			✓
Russian Federation		✓		✓
Saudi Arabia	GRM			
South Africa		✓		✓
South Korea		✓	✓	✓
Turkey				✓
United Kingdom	GRM, DR	✓		✓
United States of America		✓		✓

According to our data, some G20 countries such as the UK and France, have been engaged in green finance policymaking since the early 2000s, with the adoption of green finance principles and climate-related disclosure requirements for non-financial institutions, pension funds, and insurance companies.^6^ Indonesia stands as the earliest adopter of CRFPs; already in 1998, it started to require banks to conduct environmental impact assessments for large or high-risk loans (GoI 1998), while China and Brazil rise as the most engaged G20 countries in climate-related financial policymaking; at the end of 2017, they have both 11 policies. China adopted measures explicitly aimed at governance and risk management, such as the Green Credit Policy approved in 2007.^7^ Moreover, it encourages climate-related stress testing (CRST) at the portfolio level since 2012 with the Green Credit Guidelines.^8^ Brazil, instead, promotes sustainable development through lines of credit and programs since 2008. These policies aim to foster the population’s standards of living and environmental protection (Berchin et al. 2018). Other policies implemented in Brazil are Resolution 4.327/2014, which legally mandates that financial institutions develop and implement a social and environmental policy, and sustainability risk management and ESG disclosures. Moreover, the Brazilian Central Bank (BaCen) requires that financial institutions demonstrate how they are assessing the risk of exposure to socio-environmental damages in their assessment processes and in their calculation of the capital needed for dealing with risks.^9^ Climate-related disclosure requirements for banks have been promoted by the Chinese macroprudential authority, the Indonesian Central Bank, and Turkey’s and Mexico’s banking associations. Instead, climate-related disclosure requirements for non-financial institutions, pension funds, insurance companies, and “green” finance principles and guidelines have been widely adopted in the past 20 years in most G20 countries. These measures aim not to be macroprudential but to create a financial market aligned with climate change concerns. Notably, China, which represents a very engaged country in green finance and financial regulation, is the only jurisdiction among the G20s that has adopted differentiated reserve requirements. Finally, we note that all countries except Saudi Arabia have developed green market-shaping policies and adopted disclosure requirements for non-financial firms, insurance companies, or institutional investors.

Regarding credit allocation to priority and environmentally friendly sectors, such as green lending quotas and concessional loans, we found that they are adopted mainly by emerging economies, namely, Brazil, China, India, and Indonesia. Among developed countries, two exceptions are represented by Japan^10^ and South Korea.^11^

To account for lags between the date of adoption and the effects created by the policies, we aggregate the annual impact of climate-related financial policies in two different variables, i.e., the stock of short-term policies (STFP) and the stock of long-term policies (LTFP).^12^ The former refers to policies adopted between 2016 and 2017, the latter to policies adopted between 2000 and 2015. Both variables are then multiplied by the time-varying World Governance Indicator for Regulatory Quality from the World Bank database (Kaufmann et al. 2011) to account for the role played by institutional governance. This allows us to model the evidence that financial policies implemented in countries with a higher ability to formulate and implement sound policies and regulations are more effective.

### Other variables

The statistical model is completed by a set of control variables on economic factors, financial development, and financial stability. Regarding the former, GDP controls for the possibility of an EKC (Stern 2004) and population controls for changes in the countries’ structure that may affect the emissions profile (Meyerson 1998). Both variables are retrieved from the World Bank’s World Development Indicators database. Summary statistics show that the sample is very heterogeneous concerning these variables. Differences in GDP are relatively high and similar to CO_2_ emission distribution; it shows a positive skewness and high kurtosis. Fossil fuel consumption (TWH per capita) data are retrieved from the World Bank database. This variable serves as an indicator of the economy’s climate footprint since fossil energy consumption is a major source of environmental degradation as it contributes to polluting emissions (Meyerson 1998; Zhou and Feng 2017; Peters et al. 2020). The highest fossil fuel consumption can be observed in the USA, Saudi Arabia, and Canada. In contrast, less advanced economies like Indonesia and India tend to consume fewer fossil fuels per capita. However, since energy consumption and economic growth alone may not explain CO_2_ emissions, we need to consider other variables associated with carbon emissions. Therefore, we also include variables related to financial development, openness, and efficiency. Domestic credit to the private sector (as a share of GDP) is employed to account for the development of the financial industry; the higher the value of this indicator, the more mature the financial system. The choice to include domestic credit to the private sector is related to the evidence that economic development is a complex process that causes a structural change. In turn, a significant structural change that accompanies economic development is the financial sector’s size and structure. Return on assets (ROA) is considered to account for the banking system’s profitability and efficiency, as ROA is a good proxy for the financial system’s soundness. A high value indicates profitability and efficiency, as the banks’ profits should reduce the extent of risk in the financial market; a low value indicates the banking sector’s fragility. Overall, a better-developed financial system is believed to increase economic efficiency (Fase and Abma 2003; Sahay et al. 2015) which in turn could be associated with environmental degradation. Finally, to control the effects of financial openness, the Chinn-Ito Index (or Kaopen index) is used (Chinn and Ito 2010).^13^ It is one of the most commonly used indices in the literature (Ozturk and Acaravci 2013; You et al. 2015; Rasoulinezhad and Saboori 2018) and is constructed based on the data from the IMF Annual Report on Exchange Arrangements and Exchange Restrictions. It ranges between − 2.66 (full capital controls) and 2.66 (complete liberalization).

## Method

We first estimate our model by using pooled Ordinary Least Squares (OLS), Fixed Effects Ordinary Least Squares OLS (FE-OLS), and Dynamic Ordinary Least Squares (DOLS) estimators. However, due to the limitations of OLS estimation methods (Koenker and Bassett Jr 1978; Canay 2011; You et al. 2015), a panel quantile regression technique was employed to examine the distributional and heterogeneous effect of the different factors across CO2 emission quantiles.^14^ In particular, our econometric model specification involves the use of the Method of Moments Quantile Regression (MMQR) developed by Machado and Silva (2019). This choice is motivated by the fact that this method allows us to identify the conditional heterogeneous covariance effects of the determinants of CO_2_ emissions and provides estimators that are robust to outliers in the dependent variable. More precisely, this method allows the individual effects to affect the entire distribution, rather than being just location (means) shifters, as in the case of Koenker (2004). Moreover, the MMQR estimation technique is particularly relevant in scenarios where the panel data model is embedded with individual effects, as in our analysis.

The estimation of the conditional quantiles *Q*_*Y*_ (*τ|X*) for a model of the location/scale variant of quantile regression takes the following form:

1 $$ {Y}_{it}={a}_i+{X}_{it}^{\prime}\beta +\left({\delta}_i+{Z}_{it}^{\prime}\gamma \right){U}_{it} $$

where the probability *P*{*δ*_*i*_ + *Z*^*'*^_*it*_*γ* > o} = 1. (*a*, *β*^*'*^, *δ*, *γ*^*'*^)^*'*^ are parameters to be estimated. In particular, (*a*_*i*_, *δ*_*i*_), *i* = 1, …, *n* designates the individual *i* fixed effects and *Z* is a *k*-vector of identified components of *X* which are differentiable transformations with elements *l* given by

2 $$ {Z}_l={Z}_l(X).l=a,\dots, k $$

*X*_*it*_ is independently and identically distributed for any fixed *i* and is independent across time *t*. *U*_*it*_ is independently and identically distributed across individuals *i* and through time *t* and is orthogonal to *X*_*it*_ and normalized to satisfy the moment conditions in Machado and Silva (2019). Equation 1 implies

3 $$ {Q}_Y\left(\tau \left|X\right.\right)=\left({a}_i+\delta iq\left(\tau \right)\right)+{X}_{it}^{\prime}\beta +{Z}_{it}^{\prime}\gamma q\left(\tau \right) $$

*Q*_*Y*_ (*τ|X*) indicates the quantile distribution of the dependent variable *Y*_*it*_ that in our case is the logarithm of CO_2_ emissions per capita and is conditional on the location of independent variable *X*_*it*_. *α*_*i*_ + *δ*_*i*_*q*(*τ*) is the scalar coefficient which is indicative of the quantile-*τ* fixed effect for individual *i*. It is important to note that the individual effect in this context does not denote an intercept shift, but they are time-invariant parameters whose heterogeneous impact are allowed to differ across the quantiles of the conditional distribution of the endogenous variable *Y*. *q*(*τ*) denotes the *τ*th sample quantile which is estimated by solving the following optimization problem

4 $$ \underset{q}{\mathit{\min}}\sum \limits_i\sum \limits_t{\rho}_{\tau}\left({R}_{it}-\left({\delta}_i+{Z}_{it}^{\prime}\gamma \right)q\right) $$

where *ρ*_*τ*_ (*A*) denotes the check function.

In Eq. 3, *X*^*'*^ is a vector of independent variables as specified in Eq. 5.

5 $$ {\displaystyle \begin{array}{c}{Q}_{CO{2}_{it}}\left(\tau k\left|{\alpha}_i{x}_{it}\right.\right)=\left({\alpha}_i+{\delta}_iq\left(\tau \right)\right)+{\beta}_{1\tau }{GDP}_{it}+{\beta}_{2\tau }{GDP}_{it}^2+P{op}_{it}\\ {}+{\beta}_{4\tau }{LTFP}_{it}+{\beta}_{5\tau }{STFP}_{it}+{\beta}_{6\tau }{Domcredit}_{it}+{\beta}_{7\tau }{ChinnIto}_{it}\\ {}+{\beta}_8{ROA}_{it}+{\beta}_{9\tau }{FFC}_{it}+{Z}_{it}^{\prime}\gamma q\left(\tau \right)\end{array}} $$

where GDP denotes gross domestic product in country *i* in period *t* and Pop is the population size. LTFP represents long-term financial policies, whereas short-term financial policies are labeled as STFP. Domestic credit, Chinn-Ito Index for financial openness, return on assets, and fossil fuel consumption are denoted with Domcredit, ChinnIto, ROA, and FFC, respectively. Details about the description and source of variables are reported in the “Data” section.

## Empirical results and discussions

### Diagnostics

Before estimating the model, some standard preliminary tests are undertaken in order to verify the time-series properties of the selected variables.

First, the Pesaran (2004) cross-sectional dependence (CD) test is applied.^15^ Results are reported in Table 5 and show that the null hypothesis of strict cross-sectional independence is rejected for all variables, except for the financial openness index.

**Table 5:** Cross-sectional dependence

Variable	CD test	*p* value	Average joint *T*	Mean *ρ*	Mean abs (*ρ*)
CO2 emissions in metric tons in million	4.439	0.000	18.00	0.08	0.74
GDP (in billion current US-dollar)	46.162	0.000	18.00	0.83	0.83
Squared GDP (in billion current US-dollar)	43.251	0.000	18.00	0.78	0.78
Population, in million	32.83	0.000	18.00	0.59	0.81
Stock of long-term financial policies weighted by regulatory quality	6.075	0.000	18.00	0.11	0.53
Stock of short-term financial policies weighted by regulatory quality	2.718	0.007	18.00	0.05	0.43
Domestic credit to private sector (share of GDP)	13.079	0.000	15.14	0.26	0.60
Chinn-Ito index	0.081	0.936	18.00	0.00	0.07
Bank return on assets (percentages, after tax)	7.368	0.000	18.00	0.13	0.29
Fossil fuel consumption in TWH per capita	1.825	0.068	18.00	0.03	0.69

Second, we check the stationary properties for all variables. We employ the Augmented Dickey-Fuller (ADF) test as the most widely used procedure to examine the stationarity of a time series. We further employ the Breitung and Das (2005) panel unit root tests that assume a common autoregressive parameter for all individuals in the panel^16^ and controls for cross-sectional dependence by subtracting the cross-sectional averages from the examined series. Table 6 presents the results of the panel unit root tests, indicating that the null hypothesis of the existence of a unit root could not be rejected for all of the variables at the selected level. However, the unit root null hypothesis for all of the variables at first differences could almost be completely rejected at the 1% level. This implies that the empirical analysis should use variables in first differences; otherwise, the OLS estimation results may be biased due to a spurious relationship. The use of variables expressed in first differences would solve this problem, but we note that the long-term relationship between the variables would be lost. As we are especially interested in the long-run effects of climate-related financial policies on the distribution of CO2 emissions, first differencing is not a viable option. Nevertheless, the regression of nonstationary data may lead to spurious regression results. Therefore, in a third step, we perform a cointegration analysis to examine whether a long-run equilibrium characterizes the variables that compose the panel. If so, regression techniques can be applied to yield consistent estimates, although the data is nonstationary. The Pedroni (2004), Kao (1999) and Westerlund (2007) cointegration tests are performed, and the Westerlund variance ratio is also applied to control for cross-sectional dependence. Results are displayed in Table 7. Overall, tests’ results provide a robust indication that the data is integrated of order one. In line with existing literature adopting a similar panel quantile regression approach (see, e.g., Ike et al. 2020; An et al. 2021; Aziz et al. 2021),^17^ our analysis considers variables in levels.^18^

**Table 6:** Panel unit root tests

Variable	CO2	GDP	Pop	LTFP	STFP	TFP	ChinnIto	ROA	FFC	Domcredit
Level:										
Dickey-Fuller	2.6293	0.8353	1.5279*∗∗∗*	− 1.0265	2.9424	3.9435	− 0.1538	− 5.0747*∗∗∗*	2.1576	− 1.3782*∗*
Breitung and Das (2005)	7.3301	7.6625	13.6011	− 6.0036*∗∗∗*	1.9820	7.5366	− 0.2832	− 2.9782*∗∗∗*	4.3267	–
First differences:										
Dickey-Fuller	− 8.4972*∗∗∗*	− 7.6186*∗∗∗*	− 4.6648*∗∗∗*	− 2.8496*∗∗∗*	− 8.3703*∗∗∗*	− 5.4032*∗∗∗*	− 4.379*∗*	− 11.6094*∗∗∗*	− 7.3346*∗∗∗*	− 6.0571*∗∗∗*
Breitung and Das (2005)	− 7.8281*∗∗∗*	− 7.1616*∗∗∗*	3.6636	− 12.1130*∗∗∗*	− 4.0171*∗∗∗*	− 9.2473*∗∗∗*	− 6.3077*∗∗∗*	− 3.6171*∗∗∗*	− 9.3693*∗∗∗*	–

*p < 0*.*10, *∗∗p <* 0*.*05, *∗∗∗p <* 0*.*01

**Table 7:** Cointegration test results

Test		Test statistic	*p* value
Pedroni	Modified Phillips-Perron	2.8730	0.0020
	Phillips-Perron	− 7.6372	0.0000
	Augmented Dickey-Fuller	− 7.5883	0.0000
Kao	Modified Dickey-Fuller	− 0.2299	0.4091
	Dickey-Fuller	− 2.4571	0.0070
	Augmented Dickey-Fuller	− 2.2044	0.0137
	Unadjusted modified Dickey-Fuller	1.2270	0.1099
	Unadjusted Dickey-Fuller	− 1.5580	0.0596
Westerlund	Variance ratio	− 1.3727	0.0849

### Panel quantile regression results

In this section, we discuss the estimation results of the model specification involving the MMQR.

**Fig. 3:**
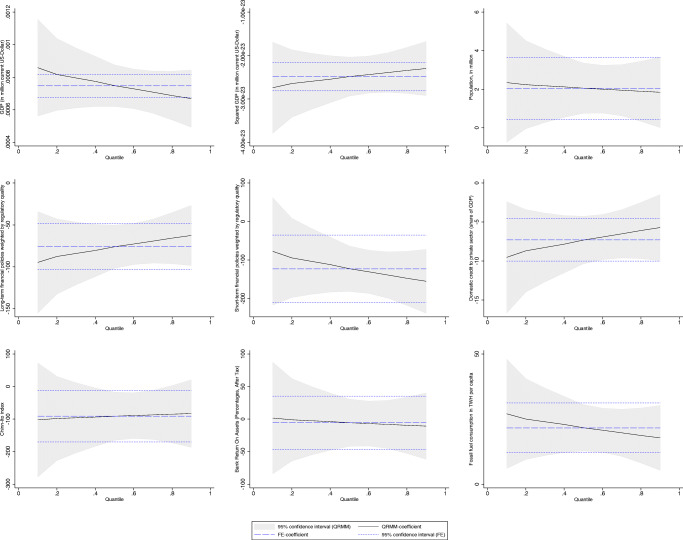
Method of Moments Panel Quantile Regression results

To allow for a comparison, we report standard OLS and the MMQR estimation results in separate tables. Table 8 shows results considering pooled OLS estimation with robust standard errors (column 1), fixed effects (F.E.) with robust standard errors (column 2), and with Discroll and Kraay standard errors (D-K S.E.) (column 3). Column 4 displays DOLS results. OLS results are also displayed in Fig. 3 (see blue dotted lines). The FE-OLS regression is augmented with Discroll and Kraay standard errors, which are more robust in the presence of cross-sectional dependence. As explained in the “Diagnostics” section, unit root and cointegration tests suggest that our data is non-stationary and integrated of order 1. Thus, coefficients are estimated consistently, but *t*-statistics may not be reliable. Consequently, we also apply the DOLS estimator proposed by Kao and Chiang (2001) for panel data settings. In the presence of non-stationarity and cointegration, the DOLS technique proposes to include lags and leads of the regressors in first differences to yield a consistent estimator with reliable *t*-statistics.^19^ We note that all estimation approaches in general yield similar results.^20^ The estimated coefficients are similar to those observed in the other three estimations procedures, except for bank return on assets. As the time series for short-term financial policy is very short by construction, there is too little variation which yields a coefficient of zero. Moreover, as *t*-statistics between fixed effects and DOLS approaches do not deviate substantially, we conclude that *t*-statistics of OLS, F.E., and F.E. (D-K S.E.) regressions are reliable, although the data is non-stationary. However, as pointed out in the methodological section, estimations performed employing the OLS approach—focusing on the mean effects—describe only a partial picture of the empirical relationship between the variables. By adopting the MMQR approach, the results provide a detailed description throughout the conditional distribution, especially in the countries with the most and least emissions. Moreover, we distinguish between the effects of the stock of recent (short-term) and older (long-term) climate-related financial policies. The MMQR estimation results are reported for the 10th, 20th, 30th, 40th, 50th, 60th, 70th, 80th, and 90th percentiles of the conditional CO_2_ emission distribution. The lower quantiles include Argentina, Turkey, Australia, Brazil, France, Indonesia, Italy, and South Africa. The higher quantiles refer to countries with higher CO_2_ emissions, such as Germany, Japan, the Russian Federation, India, the USA, and China (see Fig. 1 for more insights on the CO_2_ distribution).

**Table 8:** Climate-related financial policies and their effects on CO2 emissions: OLS estimation results

	(1) Pooled OLS	(2) FE	(3) FE (D-K S.E.)	(4) DOLS
GDP (in billion current US-dollar)	0.340 (0.0274)	0.276 (0.161)	0.276*∗∗∗* (0.0368)	0.252 (0.0552)
Population, in million	2.573*∗∗∗* (0.273)	5.225*∗* (2.548)	5.225*∗∗∗* (0.251)	5.510*∗* (2.962)
Long-term financial policies weighted by regulatory quality	− 78.69*∗∗∗* (19.40)	− 45.16 (33.30)	− 45.16*∗* (22.83)	− 68.53*∗* (37.56)
Short-term financial policies weighted by regulatory quality	− 80.82*∗∗* (31.92)	− 9.687 (85.57)	− 9.687 (23.65)	0 (99.52)
Chinn-Ito index	− 205.3*∗∗∗* (29.37)	− 67.57 (78.79)	− 67.57*∗∗* (25.79)	− 155.7*∗* (90.03)
Bank return on assets (percentages, after tax)	47.85 (34.71)	− 5.568 (40.05)	− 5.568 (11.18)	99.88*∗∗* (46.73)
Fossil fuel consumption in TWH per capita	15.27*∗∗∗* (1.248)	51.22 (34.16)	51.22*∗∗∗* (5.099)	13.57 (15.54)
Domestic credit to private sector (share of GDP)	2.310*∗∗∗* (0.836)	− 3.799 (3.671)	− 3.799*∗∗* (1.400)	
Constant	− 724.0*∗∗∗* (99.52)	− 2004.2*∗∗∗* (1307.2)	− 2004.2*∗∗∗* (120.8)	
*N*	312	312	312	247
*R*^2^*a*	0.862	0.620	0.629	0.431

Standard errors in parentheses

*p < 0*.*10, *∗∗p <* 0*.*05, *∗∗∗p <* 0*.*01

The quantile regression results based on Eq. 5 are shown in Table 9. Figure 3 shows the coefficients across all quantiles and the corresponding 95% confidence interval for all independent variables. In general, the signs and significance of the MMQR coefficients correspond to those observed in the pooled OLS, FE-OLS, and DOLS estimations.

**Table 9:** Climate-related financial policies and their effects on CO2 emissions: panel quantile regression results

	(1)	(2)	(3)	(4)	(5)	(6)	(7)	(8)	(9)
GDP (in billion current US-dollar)	0.858*∗∗∗* (0.152)	0.817*∗∗∗* (0.112)	0.795*∗∗∗* (0.0940)	0.774*∗∗∗* (0.0786)	0.749*∗∗∗* (0.0655)	0.729*∗∗∗* (0.0621)	0.709*∗∗∗* (0.0663)	0.688*∗∗∗* (0.0768)	0.669*∗∗∗* (0.0898)
Squared GDP (in billion current US-dollar)	− 0.0000274*∗∗∗* (0.00000537)	− 0.0000264*∗∗∗* (0.00000397)	− 0.0000260*∗∗∗* (0.00000332)	− 0.0000255*∗∗∗* (0.00000276)	− 0.0000249*∗∗∗* (0.00000230)	− 0.0000244*∗∗∗* (0.00000218)	− 0.0000239*∗∗∗* (0.00000233)	− 0.0000234*∗∗∗* (0.00000271)	− 0.0000230*∗∗∗* (0.00000318)
Population, in million	2.342 (1.579)	2.232*∗* (1.164)	2.176*∗∗* (0.972)	2.121*∗∗∗* (0.807)	2.053*∗∗∗* (0.668)	2.001*∗∗∗* (0.635)	1.948*∗∗∗* (0.680)	1.893*∗∗* (0.796)	1.844*∗∗* (0.937)
Long-term financial policies weighted by regulatory quality	− 94.76*∗∗∗* (30.94)	− 87.66*∗∗∗* (22.88)	− 84.02*∗∗∗* (19.11)	− 80.42*∗∗∗* (15.92)	− 76.03*∗∗∗* (13.23)	− 72.69*∗∗∗* (12.56)	− 69.22*∗∗∗* (13.43)	− 65.66*∗∗∗* (15.62)	− 62.49*∗∗∗* (18.35)
Short-term financial policies weighted by regulatory quality	− 77.81 (71.49)	− 94.67*∗* (52.73)	− 103.3*∗∗* (44.00)	− 111.8*∗∗∗* (36.57)	− 122.3*∗∗∗* (30.31)	− 130.2*∗∗∗* (28.80)	− 138.4*∗∗∗* (30.83)	− 146.9*∗∗∗* (36.01)	− 154.4*∗∗∗* (42.41)
Domestic credit to private sector (share of GDP)	− 9.542*∗∗∗* (3.652)	− 8.709*∗∗∗* (2.701)	− 8.283*∗∗∗* (2.256)	− 7.861*∗∗∗* (1.879)	− 7.346*∗∗∗* (1.562)	− 6.954*∗∗∗* (1.483)	− 6.548*∗∗∗* (1.585)	− 6.130*∗∗∗* (1.844)	− 5.759*∗∗∗* (2.165)
Chinn-Ito index	− 102.3 (89.33)	− 98.02 (65.91)	− 95.83*∗* (55.02)	− 93.67*∗∗* (45.71)	− 91.02*∗∗* (37.87)	− 89.01*∗∗* (36.02)	− 86.92*∗∗* (38.54)	− 84.77*∗* (45.03)	− 82.86 (52.99)
Bank return on assets (percentages, after tax)	1.713 (43.85)	− 1.041 (32.34)	− 2.451 (27.00)	− 3.845 (22.43)	− 5.549 (18.58)	− 6.845 (17.67)	− 8.189 (18.91)	− 9.569 (22.10)	− 10.80 (26.01)
Fossil fuel consumption in TWH per capita	27.04*∗∗* (10.72)	25.01*∗∗∗* (7.919)	23.97*∗∗∗* (6.612)	22.95*∗∗∗* (5.500)	21.70*∗∗∗* (4.563)	20.74*∗∗∗* (4.336)	19.75*∗∗∗* (4.638)	18.74*∗∗∗* (5.408)	17.84*∗∗∗* (6.360)
*N*	312	312	312	312	312	312	312	312	312

Standard errors in parentheses

*p < 0*.*10, *∗∗p <* 0*.*05, *∗∗∗p <* 0*.*01

Overall, the empirical results illustrate that the impact of the different variables included in the analysis is heterogeneous and indicate several important findings.

**Fig. 4:**
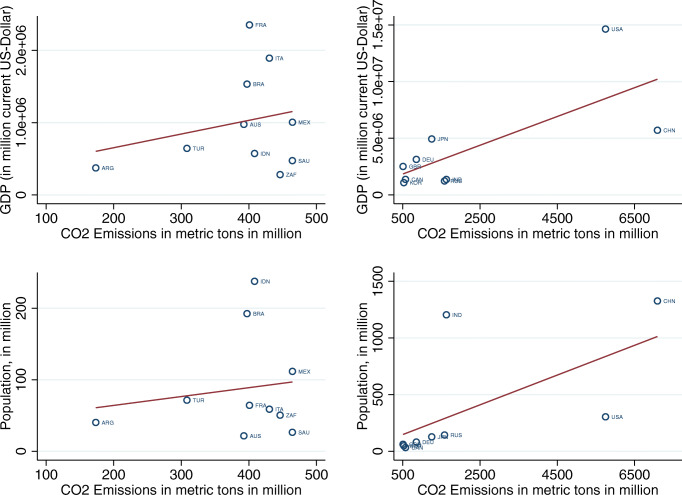
Average population, GDP, and CO2 emission in G20 by country (2000–2017)

**Fig. 5:**
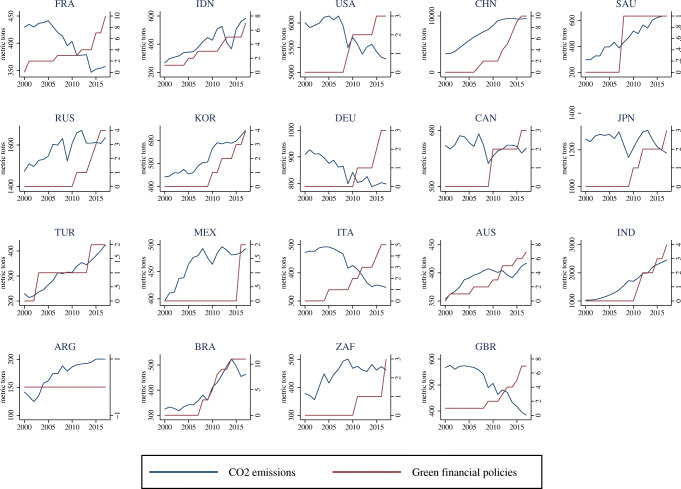
CO2 emissions by country and adoption of climate-related financial policies (2000–2017)

First, they suggest that adopting a climate-related financial policy has an overall statistically significant negative effect on CO_2_ emissions over both the short and the long term, thus implying that implementing climate-related financial policies improves the environmental quality in these countries. This result is a relevant contribution to the existing literature as it emphasizes the existence of a significant relationship between CO_2_ emissions and climate-related financial policies for the first time. In our view, our results provide a clear indication of the important role played by financial policies aligned with a climate objective, besides the “more direct” role of climate mitigation policies (Eskander and Fankhauser 2020; Le Qúeŕe et al. 2019). We note that existing global commitments imply a massive transformation in the structure of global economic activity through changes in relative prices and large-scale public and private investments (IPCC 2018; Carney 2021), thus requiring complementarities between different policy areas (Krogstrup and Oman 2019). Moreover, we note that this complementarity could be particularly relevant considering the evidence that CO2 emissions are growing regardless of the considerable efforts put forward at the international level through climate policies (Peters et al. 2020) (Fig. 4). Observing the different timing of the policies reflected in the long-term and short-term stocks, we find that the impact of long-term financial policies on CO_2_ emissions is significantly negative (at 1% level) in all quantiles, while different effects are observed for short-term policies. For example, at the 10th quantile, the coefficient of the short-term stock (STFP) is negative but insignificant, indicating that these policies do not affect the environmental quality in countries with lower emissions. The rationale is that the majority of the countries which are located at the bottom of the CO_2_ distribution adopted the bulk of the CRFPs before 2015. Indeed, as discussed in the “Climate-related financial policies in the G20” section, in countries like Australia, Brazil, France, and Indonesia, the adoption of CRFPs dates back to the early 2000s (see also the evidence reported in Figs. 2 and 5). Additionally, we observe that their impact decreases in magnitude as we move from lower to higher quantiles of the distribution. The rationale for this is to be found in the type of policies adopted. In the short term (i.e., the period 2016–2017 in our analysis), the majority of the countries located at the top of the distribution have adopted only “soft” measures, such as “green finance principles” that are usually not binding for financial institutions or disclosure requirements aimed at companies or non-financial institutions. Interestingly, when considering the long-term stock of policies, we note that their impact increases when moving from lower to higher emission countries. In this case, the effect can be explained by combining the information on the total stock of policies and types. Specifically, (i) the countries located at the top of the distribution adopted the bulk of the policies before 2015 and (ii) they adopted mandatory prudential requirements and/or promotional credit measures, which contributed to improving environmental quality in these countries characterized by higher emissions.

Second, the effect of our indicator for financial development, i.e., domestic credit to the private sector, on CO_2_ emissions is negative across all quantiles at a 1% significance level. This result confirms that an increase in private credit contributes to improving environmental quality and aligns with a stream of research that points to a positive role played by financial development in decreasing environmental degradation. Indeed, as extensively discussed in the “Literature review” section, existing literature explains that financial development amplifies investments in modern technology that may reduce carbon emissions (see Shahbaz et al. 2016; Zaidi et al. 2019; Zafar et al. 2019, among others). Moreover, we argue that our findings on financial development are related to the overall climate-related financial landscape, i.e., green finance and policy developments characterizing G20s. As discussed in previous sections, our investigation of climate-related financial policies reveals a major commitment to promoting sustainability and ecological investments in the past decades, which in turn “feeds-in” the overall financial development. Finally, we note that the impact of financial development increases as we move towards the top of the distribution. This result is consistent with the evidence that more mature financial systems characterize these countries; this encourages green technology progress and hence alleviates CO_2_ emissions.

Third, regarding financial openness, negative signs characterize all quantiles, thus confirming the hypothesis that an improvement in financial infrastructure (based on the openness of capital account) may contribute to the efficient technological use and, therefore, affect the environmental degradation as well (Tamazian et al. 2009; Tamazian and Rao 2010; Jalil and Feridun 2011; You et al. 2015). However, heterogeneous effects are observed with respect to the coefficients for the 10th, 20th, and 90th quantiles that are not significant. On the one hand, this implies that the degree of financial openness is associated with environmental degradation. On the other hand, this effect is not observed in countries with the highest and lowest emissions. Moreover, we note that significant coefficients are found in quantiles mainly referring to high- and upper-middle-income countries.

Fourth, statistically significant results cannot be reported for all quantiles regarding the indicator for financial soundness, i.e., bank return on assets. Therefore, our analysis cannot support the hypothesis that a financially sound banking system is associated with environmental degradation (Kim et al. 2020).

Fifth, the estimated coefficients on GDP are positive and significant for all quantiles and all specifications. Moreover, the squared term—that is introduced to capture the non-linear effects of economic growth on carbon emissions—is negative, proving the EKC’s existence, as discussed in the “Literature review” section. The estimated coefficients for the variable *population* are positive and statistically significant for all quantiles—except the 10th quantile. The levels of significance vary across quantiles and are higher in the middle of the distribution. This result suggests that a larger population size could harm the environment’s quality and aligns with existing empirical evidence provided by Sadorsky (2014), Yeh and Liao (2017), and Dong et al. (2018).

Finally, regarding the effects of fossil fuel consumption, the estimated coefficients show a positive effect (at 5% and 1% level) on CO_2_ emissions. This confirms the evidence that CO_2_ emissions increase due to primary energy consumption (Acaravci and Ozturk 2010; Pao and Tsai 2011; Sheraz et al. 2021). Moreover, the observed impact is larger for lower quantiles and progressively decreases as we move towards higher quantiles.

### Alternative model specifications and robustness checks

To gain further insights into our research question, we run five additional specifications of the model and check for the robustness of our findings along several dimensions. Specification I does not consider regulatory quality in implementing long-run and short-run policies; rather, regulatory quality enters the regression directly as an explanatory variable; results for this specification are shown in Table 10. Specifications II and III exclude the recent and older stocks of policies and consider the cumulated stock instead, i.e., the total stock of policies over 2000–2017, without any distinction between the long and short term. Moreover, the specifications distinguish the consideration (Specification II) or exclusion (Specification III) of the implementation’s regulatory quality. Results are reported in Tables 11 and 12 respectively. Specification IV uses the climate-related financial policy index (CRFPI) developed in D’Orazio and Thole (2021).^21^ Results are reported in Table 13.

**Table 10:** Robustness analysis: long-term stock and short-term stock of policies

	(1)	(2)	(3)	(4)	(5)	(6)	(7)	(8)	(9)
GDP (in billion current US-dollar)	0.854*∗∗∗* (0.302)	0.827*∗∗∗* (0.238)	0.806*∗∗∗* (0.190)	0.788*∗∗∗* (0.149)	0.774*∗∗∗* (0.121)	0.755*∗∗∗* (0.0910)	0.741*∗∗∗* (0.0809)	0.724*∗∗∗* (0.0877)	0.710*∗∗∗* (0.107)
Squared GDP (in billion current US-dollar)	− 0.0000270*∗∗* (0.0000107)	− 0.0000265*∗∗∗* (0.00000845)	0.0000261*∗∗∗* (0.00000675)	− 0.0000258*∗∗∗* (0.00000528)	− 0.0000255*∗∗∗* (0.00000430)	− 0.0000252*∗∗∗* (0.00000323)	− 0.0000249*∗∗∗* (0.00000287)	− 0.0000246*∗∗∗* (0.00000311)	− 0.0000243*∗∗∗* (0.00000381)
Population, in million	2.879 (2.947)	2.738 (2.320)	2.630 (1.853)	2.532*∗* (1.449)	2.460*∗∗* (1.178)	2.359*∗∗∗* (0.883)	2.286*∗∗∗* (0.784)	2.198*∗∗∗* (0.852)	2.123*∗∗* (1.044)
Long-term financial policies	− 61.45 (62.74)	− 57.83 (49.39)	− 55.05 (39.45)	− 52.52*∗* (30.86)	− 50.65*∗∗* (25.10)	− 48.06*∗∗* (18.84)	− 46.18*∗∗∗* (16.74)	− 43.92*∗∗* (18.17)	− 41.98*∗* (22.25)
Short-term financial policies	− 27.82 (143.0)	− 42.80 (112.6)	− 54.31 (89.90)	− 64.79 (70.33)	− 72.51 (57.19)	− 83.21*∗* (42.92)	− 91.00*∗∗* (38.12)	− 100.4*∗∗* (41.38)	− 108.4*∗∗* (50.70)
Regulatory Quality (Estimate)	296.7 (524.9)	223.9 (413.3)	167.9 (330.1)	117.0 (258.4)	79.46 (210.2)	27.44 (158.1)	− 10.42 (140.4)	− 55.91 (152.2)	− 94.96 (186.3)
Domestic credit to private sector (share of GDP)	− 7.128 (6.871)	− 6.964 (5.409)	− 6.837 (4.319)	− 6.723*∗∗* (3.379)	− 6.638*∗∗* (2.747)	− 6.521*∗∗∗* (2.062)	− 6.435*∗∗∗* (1.832)	− 6.333*∗∗∗* (1.989)	− 6.244*∗∗* (2.436)
Chinn-Ito index	− 155.8 (173.9)	− 144.7 (137.0)	− 136.1 (109.4)	− 128.3 (85.56)	− 122.6*∗* (69.59)	− 114.6*∗∗* (52.25)	− 108.8*∗∗* (46.43)	− 101.8*∗∗* (50.40)	− 95.84 (61.70)
Bank return on assets (percentages, after tax)	− 11.66 (85.94)	− 13.26 (67.66)	− 14.49 (54.02)	− 15.61 (42.25)	− 16.44 (34.35)	− 17.59 (25.77)	− 18.42 (22.89)	− 19.42 (24.87)	− 20.28 (30.46)
Fossil fuel consumption in TWH per capita	30.57 (22.19)	29.43*∗* (17.47)	28.55*∗∗* (13.95)	27.75*∗∗* (10.91)	27.16*∗∗∗* (8.876)	26.34*∗∗∗* (6.662)	25.74*∗∗∗* (5.919)	25.03*∗∗∗* (6.426)	24.41*∗∗∗* (7.869)
*N*	312	312	312	312	312	312	312	312	312

Standard errors in parentheses

*p < 0*.*10, *∗∗p <* 0*.*05, *∗∗∗p <* 0*.*01

**Table 11:** Robustness analysis: cumulated stock of policies

	(1)	(2)	(3)	(4)	(5)	(6)	(7)	(8)	(9)
GDP (in billion current US-dollar)	0.833*∗∗∗* (0.211)	0.815*∗∗∗* (0.163)	0.803*∗∗∗* (0.132)	0.793*∗∗∗* (0.107)	0.780*∗∗∗* (0.0829)	0.770*∗∗∗* (0.0719)	0.760*∗∗∗* (0.0718)	0.752*∗∗∗* (0.0796)	0.742*∗∗∗* (0.0974)
Squared GDP (in billion current US-dollar)	− 0.0000259*∗∗∗* (0.00000745)	− 0.0000257*∗∗∗* (0.00000574)	− 0.0000256*∗∗∗* (0.00000466)	− 0.0000255*∗∗∗* (0.00000378)	− 0.0000254*∗∗∗* (0.00000292)	− 0.0000254*∗∗∗* (0.00000253)	− 0.0000253*∗∗∗* (0.00000253)	− 0.0000252*∗∗∗* (0.00000281)	− 0.0000251*∗∗∗* (0.00000344)
Population, in million	3.828*∗* (2.243)	3.457*∗∗* (1.730)	3.208*∗∗* (1.403)	2.989*∗∗∗* (1.140)	2.724*∗∗∗* (0.880)	2.521*∗∗∗* (0.763)	2.301*∗∗∗* (0.761)	2.140*∗∗* (0.847)	1.928*∗* (1.037)
Cumulated total financial policies	− 50.04 (38.62)	− 49.58*∗* (29.79)	− 49.26*∗∗* (24.17)	− 48.99*∗∗* (19.61)	− 48.66*∗∗∗* (15.13)	− 48.41*∗∗∗* (13.11)	− 48.13*∗∗∗* (13.11)	− 47.93*∗∗∗* (14.58)	− 47.66*∗∗∗* (17.85)
Regulatory Quality (Estimate)	341.2 (362.4)	260.5 (279.9)	206.3 (227.0)	158.6 (184.8)	101.0 (143.3)	56.75 (124.2)	9.055 (123.7)	− 26.13 (137.0)	− 72.27 (167.9)
Domestic credit to private sector (share of GDP)	− 6.429 (4.656)	− 6.183*∗* (3.592)	− 6.018*∗∗* (2.914)	− 5.872*∗∗* (2.366)	− 5.697*∗∗∗* (1.826)	− 5.562*∗∗∗* (1.583)	− 5.416*∗∗∗* (1.582)	− 5.309*∗∗∗* (1.759)	− 5.168*∗∗* (2.152)
Chinn-Ito index	− 199.5*∗* (118.3)	− 176.5*∗* (91.38)	− 161.1*∗∗* (74.12)	− 147.5*∗∗* (60.33)	− 131.2*∗∗∗* (46.77)	− 118.6*∗∗∗* (40.56)	− 105.0*∗∗∗* (40.40)	− 94.97*∗∗* (44.75)	− 81.84 (54.80)
Bank return on assets (percentages, after tax)	− 16.16 (57.18)	− 16.67 (44.10)	− 17.01 (35.78)	− 17.31 (29.03)	− 17.68 (22.39)	− 17.96 (19.41)	− 18.26 (19.41)	− 18.48 (21.59)	− 18.77 (26.42)
Fossil fuel consumption in TWH per capita	31.14*∗∗* (15.26)	29.17*∗∗* (11.78)	27.84*∗∗∗* (9.553)	26.68*∗∗∗* (7.763)	25.27*∗∗∗* (6.003)	24.19*∗∗∗* (5.205)	23.03*∗∗∗* (5.195)	22.17*∗∗∗* (5.766)	21.05*∗∗∗* (7.058)
*N*	312	312	312	312	312	312	312	312	312

Standard errors in parentheses

*p < 0*.*10, *∗∗p <* 0*.*05, *∗∗∗p <* 0*.*01

**Table 12:** Robustness analysis: cumulated stock of policies, accounting for regulatory quality

	(1)	(2)	(3)	(4)	(5)	(6)	(7)	(8)	(9)
GDP (in billion current US-dollar)	0.825*∗∗∗* (0.151)	0.794*∗∗∗* (0.118)	0.769*∗∗∗* (0.0935)	0.753*∗∗∗* (0.0799)	0.731*∗∗∗* (0.0669)	0.713*∗∗∗* (0.0629)	0.692*∗∗∗* (0.0672)	0.674*∗∗∗* (0.0773)	0.659*∗∗∗* (0.0896)
Squared GDP (in billion current US-dollar)	− 0.0000259*∗∗∗* (0.00000548)	− 0.0000252*∗∗∗* (0.00000426)	− 0.0000247*∗∗∗* (0.00000337)	− 0.0000244*∗∗∗* (0.00000288)	− 0.0000239*∗∗∗* (0.00000239)	− 0.0000236 (0.00000225)	− 0.0000232*∗∗∗* (0.00000241)	− 0.0000228*∗∗∗* (0.00000279)	− 0.0000225*∗∗∗* (0.00000324)
Population, in million	2.625*∗* (1.590)	2.496*∗∗* (1.232)	2.394*∗∗* (0.977)	2.327*∗∗∗* (0.831)	2.236*∗∗∗* (0.689)	2.164*∗∗∗* (0.647)	2.078*∗∗∗* (0.696)	2.005*∗∗* (0.809)	1.942*∗∗* (0.940)
Cumulated total financial policies weighted by regulatory quality	− 59.69*∗∗* (25.88)	− 54.79*∗∗∗* (20.11)	− 50.94*∗∗∗* (15.94)	− 48.39*∗∗∗* (13.60)	− 44.92*∗∗∗* (11.33)	− 42.22*∗∗∗* (10.65)	− 38.95*∗∗∗* (11.41)	− 36.16*∗∗∗* (13.20)	− 33.78*∗∗* (15.31)
Domestic credit to private sector (share of GDP)	− 8.931*∗∗* (3.718)	− 8.318*∗∗∗* (2.888)	− 7.835*∗∗∗* (2.290)	− 7.515*∗∗∗* (1.953)	− 7.082*∗∗∗* (1.625)	− 6.743*∗∗∗* (1.528)	− 6.333*∗∗∗* (1.638)	− 5.985*∗∗∗* (1.895)	− 5.686*∗∗∗* (2.199)
Chinn-Ito index	− 69.07 (94.17)	− 68.98 (73.02)	− 68.92 (57.89)	− 68.88 (49.28)	− 68.82^*∗*^ (40.87)	− 68.78^*∗*^ (38.40)	− 68.72^*∗*^ (41.27)	− 68.68 (47.94)	− 68.64 (55.69)
Bank return on assets (percentages, after tax)	13.40 (46.59)	8.809 (36.14)	5.190 (28.65)	2.798 (24.39)	− 0.454 (20.25)	− 2.987 (19.02)	− 6.063 (20.43)	− 8.674 (23.72)	− 10.91 (27.55)
Fossil fuel consumption in TWH per capita	26.70*∗∗* (11.26)	25.08*∗∗∗* (8.741)	23.81*∗∗∗* (6.929)	22.97*∗∗∗* (5.904)	21.82*∗∗∗* (4.907)	20.93*∗∗∗* (4.611)	19.84*∗∗∗* (4.948)	18.92*∗∗∗* (5.736)	18.14*∗∗∗* (6.661)
*N*	312	312	312	312	312	312	312	312	312

Standard errors in parentheses

*p < 0*.*10, *∗∗p <* 0*.*05, *∗∗∗p <* 0*.*01

**Table 13:** Robustness analysis: role of the climate-related financial policy index

	(1)	(2)	(3)	(4)	(5)	(6)	(7)	(8)	(9)
GDP (in billion current US-dollar)	0.538*∗∗* (0.271)	0.524*∗∗* (0.205)	0.516*∗∗∗* (0.169)	0.508*∗∗∗* (0.141)	0.501*∗∗∗* (0.122)	0.493*∗∗∗* (0.114)	0.485*∗∗∗* (0.123)	0.478*∗∗∗* (0.143)	0.469*∗∗∗* (0.180)
Squared GDP (in billion current US-dollar)	− 0.000000486 (0.0000195)	− 0.000000903 (0.0000148)	− 0.00000116 (0.0000121)	− 0.00000139 (0.0000101)	− 0.00000161 (0.00000877)	− 0.00000185 (0.00000821)	− 0.00000210 (0.00000885)	− 0.00000232 (0.0000103)	− 0.00000262 (0.0000130)
Domestic credit to private sector (share of GDP)	− 6.024 (3.889)	− 5.909*∗∗* (2.944)	− 5.837*∗∗* (2.416)	− 5.774*∗∗∗* (2.024)	− 5.713*∗∗∗* (1.750)	− 5.645*∗∗∗* (1.639)	− 5.577*∗∗∗* (1.767)	− 5.516*∗∗∗* (2.053)	− 5.434*∗∗* (2.585)
Population, in million	5.618*∗∗∗* (1.718)	5.169*∗∗∗* (1.301)	4.891*∗∗∗* (1.067)	4.647*∗∗∗* (0.897)	4.409*∗∗∗* (0.778)	4.146*∗∗∗* (0.726)	3.881*∗∗∗* (0.782)	3.642*∗∗∗* (0.908)	3.325*∗∗∗* (1.143)
Climate-related financial policy index	− 1.184*∗* (0.611)	− 1.088*∗∗* (0.463)	− 1.028*∗∗∗* (0.380)	− 0.975*∗∗∗* (0.319)	− 0.924*∗∗∗* (0.276)	− 0.867*∗∗∗* (0.258)	− 0.810*∗∗∗* (0.278)	− 0.758*∗∗* (0.323)	− 0.690*∗* (0.406)
Chinn-Ito index	− 64.79 (60.89)	− 60.20 (46.10)	− 57.36 (37.84)	− 54.86*∗* (31.70)	− 52.42*∗* (27.42)	− 49.73*∗* (25.68)	− 47.02*∗* (27.68)	− 44.57 (32.15)	− 41.33 (40.49)
Bank return on assets (percentages, after tax)	− 4.989 (31.75)	− 7.718 (24.03)	− 9.411 (19.72)	− 10.90 (16.52)	− 12.35 (14.29)	− 13.95 (13.38)	− 15.56 (14.42)	− 17.02 (16.76)	− 18.94 (21.11)
Fossil fuel consumption in TWH per capita	28.28*∗∗∗* (9.299)	26.52*∗∗∗* (7.047)	25.42*∗∗∗* (5.785)	24.46*∗∗∗* (4.860)	23.53*∗∗∗* (4.212)	22.49*∗∗∗* (3.938)	21.45*∗∗∗* (4.241)	20.51*∗∗∗* (4.921)	19.26*∗∗∗* (6.188)
*N*	294	294	294	294	294	294	294	294	294

Standard errors in parentheses

*p < 0*.*10, *∗∗p <* 0*.*05, *∗∗∗p <* 0*.*01

Finally, as a further robustness check, we control for effects of international climate mitigation policies and included the dummy variable Kyoto which measures the adoption of the Kyoto Protocol^22^ in our sample. In line with existing empirical evidence (see, e.g., Aichele and Felbermayr 2013; Iwata and Okada 2014), we found that the adoption of the Kyoto Protocol reduced CO2 emissions across the whole distribution; coefficients are higher in the lower quantiles, as shown in Table 14. Moreover, by including this control variable, we note that our results regarding the role of long-term and short-term financial policies do not change. The coefficients display significant negative signs for both long-term and short-term and are not significant for the lowest quantiles of short-term financial policies. However, we note that in this case, the coefficients are higher with respect to the baseline results described in the “Empirical results and discussions” section.

**Table 14:** Robustness analysis: role of the Kyoto Protocol adoption

	(1)	(2)	(3)	(4)	(5)	(6)	(7)	(8)	(9)
GDP (in billion current US-dollar)	0.951*∗∗∗* (0.161)	0.911*∗∗∗* (0.128)	0.869*∗∗∗* (0.0956)	0.844*∗∗∗* (0.0791)	0.815*∗∗∗* (0.0675)	0.784*∗∗∗* (0.0658)	0.769*∗∗∗* (0.0689)	0.751*∗∗∗* (0.0773)	0.730*∗∗∗* (0.0910)
Squared GDP (in billion current US-dollar)	− 0.0000310*∗∗∗* (0.00000563)	− 0.0000301*∗∗∗* (0.00000444)	− 0.0000292*∗∗∗* (0.00000330)	− 0.0000286*∗∗∗* (0.00000273)	− 0.0000280*∗∗∗* (0.00000232)	− 0.0000273*∗∗∗* (0.00000227)	− 0.0000270*∗∗∗* (0.00000239)	− 0.0000266*∗∗∗* (0.00000269)	− 0.0000262*∗∗∗* (0.00000316)
Population, in million	2.088 (1.560)	2.232*∗* (1.225)	2.382*∗∗∗* (0.908)	2.472*∗∗∗* (0.751)	2.574*∗∗∗* (0.635)	2.686*∗∗∗* (0.623)	2.738*∗∗∗* (0.661)	2.803*∗∗∗* (0.745)	2.879*∗∗∗* (0.874)
Long-term financial policies weighted by regulatory quality	− 85.31*∗∗∗* (29.13)	− 75.38*∗∗∗* (23.03)	− 65.06*∗∗∗* (17.19)	− 58.86*∗∗∗* (14.22)	− 51.83*∗∗∗* (12.13)	− 44.09*∗∗∗* (11.82)	− 40.52*∗∗∗* (12.40)	− 36.01*∗∗∗* (13.94)	− 30.77*∗* (16.43)
Short-term financial policies weighted by regulatory quality	− 53.91 (63.01)	− 57.85 (49.38)	− 61.95*∗* (36.53)	− 64.41*∗∗* (30.19)	− 67.19*∗∗∗* (25.48)	− 70.26*∗∗∗* (25.04)	− 71.68*∗∗∗* (26.68)	− 73.47*∗∗* (30.05)	− 75.55*∗∗* (35.29)
Domestic credit to private sector (share of GDP)	− 5.772 (4.022)	− 5.373*∗* (3.157)	− 4.957*∗∗* (2.339)	− 4.707*∗∗* (1.934)	− 4.424*∗∗∗* (1.634)	− 4.113*∗∗* (1.604)	− 3.969*∗∗* (1.705)	− 3.787*∗∗* (1.920)	− 3.576 (2.255)
Chinn-Ito index	− 107.3 (86.99)	− 97.95 (68.27)	− 88.17*∗* (50.58)	− 82.31*∗∗* (41.82)	− 75.65*∗∗* (35.34)	− 68.32*∗∗* (34.69)	− 64.94*∗* (36.87)	− 60.67 (41.52)	− 55.71 (48.76)
Bank return on assets (percentages, after tax)	26.05 (43.29)	26.63 (33.94)	27.24 (25.12)	27.60 (20.76)	28.02 (17.52)	28.47*∗* (17.22)	28.68 (18.34)	28.95 (20.65)	29.26 (24.25)
Fossil fuel consumption in TWH per capita	24.20*∗∗∗* (9.151)	23.81*∗∗∗* (7.176)	23.40*∗∗∗* (5.312)	23.15*∗∗∗* (4.391)	22.87*∗∗∗* (3.707)	22.56*∗∗∗* (3.642)	22.42*∗∗∗* (3.876)	22.24*∗∗∗* (4.366)	22.03*∗∗∗* (5.126)
Kyoto dummy	− 262.7*∗* (142.2)	− 273.9*∗∗* (111.5)	− 285.5*∗∗∗* (82.45)	− 292.5*∗∗∗* (68.14)	− 300.4*∗∗∗* (57.49)	− 309.1*∗∗∗* (56.51)	− 313.1*∗∗∗* (60.22)	− 318.2*∗∗∗* (67.84)	− 324.1*∗∗∗* (79.67)
*N*	312	312	312	312	312	312	312	312	312

Standard errors in parentheses

*p < 0*.*10, *∗∗p <* 0*.*05, *∗∗∗p <* 0*.*01

Overall, the results from the various alternative specifications largely support the findings discussed in the “Panel Quantile regression results” section. The effect of long-term, short-term, and cumulated financial policies, as well as the CRFP index, is negative and heterogeneous across different quantiles in the conditional distribution of CO_2_ emissions. However, in specifications I and III (i.e., those excluding the regulatory quality in the interaction term), significant effects are observed only for higher quantiles, namely, the 60th, 70th, and 80th for long-term financial policies and 60th, 70th, 80th, and 90th for short-term financial policies. Regarding the effects of the cumulated policies, they are also found to improve environmental quality across all quantiles. In specification II, the coefficients are not significant for countries located at the bottom of the distribution, i.e., those with lower emissions. This is consistent with the result discussed in “Panel Quantile regression results” section. Domestic credit to the private sector is statistically significant at almost all quantiles across all specifications and has a negative impact on CO_2_ emissions. Coefficients for other control variables are similar to the results reported above and do not seem to be sensitive to a particular estimation procedure or alternative measures of climate-related policies. Thus, we conclude that the results reported in this paper are robust. Our results also proved to be robust if another measure of financial policies is applied. Indeed, comparing the coefficients of the CRFP index to the coefficients of long-run financial policies in Table 9, we observe a similar pattern. Coefficients are negative in lower rather than higher quantiles; this supports the main finding of the empirical analysis.

## Conclusions and policy implications

This paper aimed to study the effects of financial and economic development, energy consumption, and climate-related financial policies on CO_2_ emissions in G20 countries. By focusing on the climate-related financial policy landscape developed between 2000 and 2017, we investigated the determinants of carbon emissions in G20 countries through the panel quantile regression approach developed by Machado and Silva (2019). This method takes the unobserved individual heterogeneity and distributional heterogeneity into consideration and allows us to obtain a complete understanding of the factors that affect our sample’s carbon emissions distribution.

The investigation results indicate that the impacts of various economic and financial factors on carbon emission are heterogeneous. First, we find that the hypothesis at the core of our analysis is confirmed, specifically that the implementation of climate-related financial reduces CO_2_ emissions. This result represents a relevant contribution to the existing literature as it emphasizes the existence of a significant relationship between CO_2_ emissions and climate-related financial policies for the first time. Evidence is provided for recently implemented measures (i.e., those adopted in 2016 and 2017) and policies adopted before 2016. The analysis shows that the older stock of policies has a larger impact on emissions in high emission countries, i.e., those located at the top of the distribution. Regarding more recent policies, they have larger effects on the reduction of CO_2_ emission in countries in lower—rather than upper—quantiles. However, they do not affect the environmental quality in countries located at the 10th quantile since the coefficient is not significant. Second, in line with existing literature, we find that financial development contributes to improving environmental quality, and its impact increases by moving from lower to higher emission countries. Third, further heterogeneity is observed regarding financial openness. We note that the degree of financial openness of a country is associated with environmental degradation, but this is not observed in countries with the highest and lowest emissions. Fourth, our analysis does not support the hypothesis that a financially sound banking system—as proxied by the ROA—is associated with environmental degradation. Finally, our results confirm existing literature regarding the impact of the level of GDP, population, and fossil fuel consumption on environmental degradation and the existence of the EKC relationship. These results are generally robust for alternative model specifications considering alternative variables to study climate-related financial policies and the role of the Kyoto Protocol adoption.

The empirical analysis offered in the paper is relevant because the devastating consequences of environmental degradation on humanity and economic systems represent a pressing issue for governments and societies. Indeed, according to the latest IPCC (2018) report, G20 countries need to cut their current emissions by at least 45% in 2030 (below 2010 levels) to be in line with global benchmarks set on 1.5 °C.

Several important conclusions can be drawn from our empirical analysis. First, building on the evidence that climate-related financial policies can play a role in the mitigation strategy of G20 countries, they should be more actively promoted at the global level. Second, our study suggests that countries should not also increase the number of measures implemented in this policy area but also aim for specific measures to witness environmental quality improvements. Indeed, our investigation shows that the policies’ impact on environmental quality has been larger for quantiles characterized by countries adopting mandatory prudential measures and credit allocation policies. Moreover, this effect has been observed for the long-term stock of policies. Policies implemented more recently are mostly aimed at defining criteria and guidelines for green banking rather than actively mobilizing green finance; this might explain the smaller impact on CO_2_ emissions.

Third, in our view, the evidence that shows no role—or a smaller role—of climate-related financial policies in the bottom of the CO_2_ distribution points to the need for a greater engagement of lower emission countries, such as Argentina, Turkey, and Australia, in climate-related financial policymaking to achieve their mitigation objectives.

## Data Availability

The datasets used and/or analyzed during the current study are available from the corresponding author on reasonable request.

## Footnotes

1 This objective is laid out in Article 2 of the Paris Agreement that calls for “making finance flows consistent with a pathway towards low greenhouse gas emissions and climate- resilient development” (UN-FCCC 2016).

2 Existing literature usually points to carbon pricing and fiscal policy (e.g., taxes on emissions, R&D subsidies) as the “first-best” options for climate change mitigation (Stern et al. 2006). However, according to a review of policies in the NCI (2020) database, their adoption does not seem widespread in the G20 sample. This evidence could be explained considering the debate on the introduction of carbon prices (Jakob et al. 2016; Drews and Van den Bergh 2016; Baranzini et al. 2017; Boyce 2018; Dorband et al. 2019; Rosenbloom et al. 2020). Regarding fiscal policy actions, they are usually more widespread in advanced economies, and, as pointed out in Krogstrup and Oman (2019), “they may not be enough in a world where climate distortions interact with other types of market and government failures and where political economy considerations are important” (Krogstrup and Oman 2019, p.8).

3 They aim at assessing the resilience of the financial system to adverse climate shocks by considering the possible impact of hypothetical climate-related shock scenarios on the stability of individual financial institutions and the financial system in its complexity (NGFS 2019).

4 Among EU countries, we note that France was the first country to issue a sovereign green bond in 2017; nevertheless, it does not have a sustainable finance taxonomy per se (OECD 2020).

5 The policies derive from a survey of official documents by central banks, financial supervisory authorities, governments, and banking associations. The survey and data are described in D’Orazi (2021). Overall, in our panel, 44.8% of policies are green financial principles, 31.7% are represented by other disclosure requirements, 13.2% accounts for prudential measures, and 10.3% are promotional credit measures.

6 European Central Bank (ECB) is also showing a progressively increasing commitment to “greening the financial system” by announcing the goal to make climate change a “mission-critical” priority (FT 2020). Moreover, it emphasizes that banks lag on their climate-related and environmental risk disclosures, despite some improvement since 2019 (ECB 2020).

7 It recommends banks to include environmental compliance and environmental risk assessment in the loan origination process (Wang et al. 2019; Xing et al. 2020).

8 The Network for Greening the Financial Sector (NGFS) gave an additional impulse to this trend in 2019, leading several of its members to incorporate socially responsible investing in their portfolio management and reviewing their operations (NGFS 2017, 2019). The UK government and regulators, the Central Bank of the Netherland, and the Banque de France, among others, have lately shown strong leadership in this area (Carney 2015; DNB 2017; Batten et al. 2016; Campiglio et al. 2018) and planned to conduct CRST in upcoming years.

9 See Brazilian Circular on Internal Process of Capital Adequacy Assessment, No. 3547/2011.

10 In 2010, the Bank of Japan launched the Loan Supporting program consisting of concessional loans for priority sectors (i.e., environment and energy business).

11 In 2017, the Shinhan Bank launched its “Green Management Firm Loan Program” with the South Korean Ministry of Environment and KEITI, a UNEP FI Supporting Institution. The program aims at promoting Eco-friendly Small and Medium Enterprises, and loans are created with a lower interest rate.

12 A similar approach has been developed in Eskander and Fankhauser (2020) to study the impact of national climate legislation on the reduction of GHG emissions.

13 In line with existing literature, we note that the omission of financial openness from the carbon emissions model could result in possible biased and misleading empirical results. However, we also note that the research on financial openness and its effects on CO_2_ emissions is more recent and still in its infancy.

14 The approach was first introduced by Koenker and Bassett Jr. (1978) and applied in a number of empirical applications (see, e.g., Lamarche 2010; Canay 2011; Galvao Jr 2011, for early contributions).

15 It is worth checking for cross-sectional dependence within the panel as it can distort the true parameter values of coefficient estimates. It may arise due to unobserved common factors and, if ignored, it can diminish panel data efficiency gains (Phillips and Sul 2003).

16 Breitung and Das (2005) panel unit root test requires data to be strongly balanced. Therefore, the test cannot be performed for the variable domestic credit.

17 According to a literature review on the implementation of the panel quantile regression method, most studies perform preliminary tests on the panel data and investigate cross-sectional dependence, stationarity, and cointegration to ensure the reliability of the estimation.

18 We note that although the coefficients are consistent, the *t*-values may not be interpretable. Therefore, most studies also perform a DOLS that allows estimating non-stationary but integrated data in levels. The MMQR approach by Machado and Silva (2019) is very recent, and existing theoretical literature does not deal yet with the properties and requirements of the applied data. When dealing with the estimation approach of the MMQR, existing literature usually proceeds as follows: after performing the standard diagnostics, the estimation of the unknown parameters is always carried out on variables expressed in levels if the data is non-stationary but cointegrated. To control whether *t*-values are reliable, linear regression methods that allow data to be non-stationary and cointegrated as, e.g., DOLS, are applied in a second step as a benchmark for the *t*-values estimated in the MMQR (see, among others, the applications in Ike et al. 2020; An et al. 2021; Aziz et al. 2021).

19 Fully Modified Ordinary Least Squares (FM-OLS) developed by Pedroni (2004) allows for heterogeneous serial correlation properties of the error processes across individual members of the panel. However, Kao and Chiang (2001) have shown that the DOLS estimator outperforms the FM-OLS estimator in finite samples. This motivates our choice to not include FM-OLS in our analysis (see Pedroni 2001; Breitung and Pesaran 2008, for additional analysis on the properties of DOLS and FM-OLS estimators).

20 Due to two leads and two lags of the regressors in first differences, the number of observations of the DOLS estimation is limited to 247 instead of 342 as in the other specifications. Further, we note that the DOLS approach does not compute an intercept. As it requires data to be balanced, the variable domestic credit to the private sector was excluded from the estimation.

21 This index measures countries’ engagement in climate-related financial policymaking by taking into account the bindingness of the policy, i.e., whether it is mandatory, voluntary, or not binding. It is a composite index built as a weighted sum of different climate-related policy areas, as described in the “Data” section. The rationale of the index is that its value increases with the higher involvement of the country in green financial policymaking.

22 The Kyoto Protocol was adopted on 11 December 1997 and entered into force on 16 February 2005. It operationalizes the United Nations Framework Convention on Climate Change by committing industrialized countries and economies in transition to limit and reduce greenhouse gases (GHG) emissions under agreed individual targets.
